# Elevated VMP1 expression in acute myeloid leukemia amplifies autophagy and is protective against venetoclax-induced apoptosis

**DOI:** 10.1038/s41419-019-1648-4

**Published:** 2019-05-29

**Authors:** Hendrik Folkerts, Albertus T. Wierenga, Fiona A. van den Heuvel, Roy R. Woldhuis, Darlyne S. Kluit, Jennifer Jaques, Jan Jacob Schuringa, Edo Vellenga

**Affiliations:** 1 Department of Hematology, Cancer Research Center Groningen, University Medical Center Groningen, University of Groningen, Groningen, The Netherlands; 2 Department of Laboratory Medicine, University Medical Center Groningen, Groningen, The Netherlands

**Keywords:** Acute myeloid leukaemia, Mitophagy

## Abstract

Vacuole membrane protein (VMP1) is a putative autophagy protein, which together with Beclin-1 acts as a molecular switch in activating autophagy. In the present study the role of VMP1 was analysed in CD34^+^ cells of cord blood (CB) and primary acute myeloid leukemia (AML) cells and cell lines. An increased expression of VMP1 was observed in a subset of AML patients. Functional studies in normal CB CD34^+^ cells indicated that inhibiting VMP1 expression reduced autophagic-flux, coinciding with reduced expansion of hematopoietic stem and progenitor cells (HSPC), delayed differentiation, increased apoptosis and impaired in vivo engraftment. Comparable results were observed in leukemic cell lines and primary AML CD34^+^ cells. Ultrastructural analysis indicated that leukemic cells overexpressing VMP1 displayed a reduced number of mitochondrial structures, while the number of lysosomal degradation structures was increased. The overexpression of VMP1 did not affect cell proliferation and differentiation, but increased autophagic-flux and improved mitochondrial quality, which coincided with an increased threshold for venetoclax-induced loss of mitochondrial outer membrane permeabilization (MOMP) and apoptosis. In conclusion, our data indicate that in leukemic cells high VMP1 is involved with mitochondrial quality control.

## Introduction

Macroautophagy (referred to as autophagy) is a multi-step catabolic process involved in lysosomal degradation of redundant cellular constituents, such as organelles and proteins^1–3^. Autophagy is essential for hematopoietic stem cell (HSC) maintenance, in part by actively limiting mitochondrial oxidative metabolism^4,5^. During HSC differentiation the autophagic-flux gradually declines, but autophagy might have distinct functions in terminal differentiated cells^6^. It controls the clearance of mitochondria in erythroid precursor cells and is essential for monocyte-to-macrophage differentiation^7–10^. For the malignant counterpart, studies have shown that a subgroup of AML cells heavily relies on autophagy for their survival^11–13^, whereby increased autophagy is associated with therapy resistance^14–18^. The increased autophagy is observed especially in poor-risk AML^11^. Since mutations in autophagy genes have only been observed in the minority of patients^19^, increased autophagy is most likely related to other phenomena, such as therapy-induced changes in their metabolism. Inhibition of autophagy by knockdown of essential autophagy genes such as ATG5 or ATG7 impairs AML in vitro cell proliferation and in vivo engraftment^11,16,20^. This vulnerability relies on accumulation of (dysfunctional) mitochondria, as evident by the increased reactive oxygen species (ROS) production and the activation of p53-mediated apoptosis^11^.

Vacuole membrane protein (VMP1) is an additional autophagy protein residing in the endoplasmic reticulum (ER) membrane^21,22^, which can interact with the BH3 domain of Beclin-1, thereby activating autophagy^23^. The anti-apoptotic BCL-2 family members can also bind to the BH3 domain of Beclin-1, resulting in the dissociation of VMP1 and subsequent inhibition of the autophagic-flux^23,24^. Little information is available on VMP1 in hematopoietic cells, but in solid tumors it has been shown that VMP1-dependent autophagy can be activated under stress conditions, such as starvation and hypoxia^25,26^. We therefore determined whether VMP1 is essential for autophagy in normal and malignant hematopoiesis and whether high VMP1 expression provides survival benefits for leukemic cells.

The results showed that VMP1 is important for survival of normal hematopoietic stem cells and progenitor (HSCP) cells in vitro and in vivo. Moreover, VMP1 expression was significantly increased in AMLs. Overexpression studies and ultrastructurally analysis revealed that VMP1 is involved in mitochondrial quality control, thereby protecting cells against oxidative stress. We concluded that high VMP1 expression increases autophagic flux and the threshold for venetoclax-mediated loss of MOMP and thereby reducing apoptosis-mediated cell death.

## Material and methods

### Isolation and culture of human CD34^+^ cells

Umbilical cord blood (UCB) obtained from full-term healthy neonates who were born at the Obstetrics departments of the Martini Hospital and the University Medical Center Groningen (Groningen, the Netherlands) after informed consent. AML blasts derived from peripheral blood cells or bone marrow were obtained from patients in accordance with the Declaration of Helsinki; the protocols were approved by the Medical Ethics Committee of the University Medical Center Groningen (UMCG). Mononuclear cells (MNC) were isolated from UCB, or peripheral blood or bone marrow from AML patients by Ficol density centrifugation, and CD34^+^ cells were subsequently isolated with the autoMACS pro-separator (Miltenyi Biotec, Amsterdam, the Netherlands). With this method CD34^+^ cells are purified twice in order to yield a high purity (>97%). In addition, for FACS-based experiments the CD34^+^ fraction was selected using CD34^+^ markers. AML patient characteristics are indicated in supplemental Table S1. Monoclonal antibodies CD34, CD123, MPO, CD33, HLA-Dr, CD13, CD14, CD15, CD36 were used for determination of the AML phenotype.

### Cell culture

Primary AML, normal bone marrow or CB-derived CD34^+^ cells (with a CD34^+^ purity of >97% after isolation) were cultured in suspension or in T25 flasks pre-coated with MS5 stromal cells in Gartners medium: Alpha-MEM (Lonza, Leusden, the Netherlands) supplemented with 12.5% FCS and 12.5% Horse serum (Sigma-Aldrich, Saint Louis, USA), 1% penicillin/streptomycin (PAA Laboratories, Dartmouth, USA), 1 µM hydrocortisone (Sigma-Aldrich), 57.2 μM β–mercaptoethanol, and cytokines: G-CSF, Human TPO agonist; Romiplostim (Amgen, Breda, the Netherlands) and IL-3 (20 ng/mL each). The relative increase in Cyto-ID signal after overnight incubation with 20 µM hydroxychloroquine (HCQ) was defined as the autophagy flux^6,11^. The concentration and incubation time of HCQ for measuring autophagic-flux was validated and is based on maximal accumulation of autophagosomes, without affecting cell viability, after overnight incubation with HCQ^6,11^. The leukemic cell lines HL60, OCIM3, MOLM13 and THP1 were obtained from ATCC and the cell lines were all tested mycoplasma free by PCR. All leukemic cell lines cells were cultured in RPMI 1640, supplemented with 10% FCS and 1% penicillin/streptomycin.

### Antibodies and reagents

The following anti-human antibodies were used: mouse anti-SQSTM1/p62 (sc-28359) and rabbit (sc-130656) or mouse (sc-47778) anti-Actin, from Santa Cruz (Santa Cruz, CA, USA), Mouse anti-LC3 (5F10, 0231-100) from Nanotools (Munich, Germany), P62, and anti-BCL-2 (Santa Cruz, CA, USA), anti-TOM20 and anti-VMP1 were obtained from cell signalling (Leiden, the Netherlands), Hydroxychloroquine (HCQ), was obtained from Sigma-Aldrich. Venetoclax/ABT-199 (BCL-2 inhibitor, Selleckchem Munich, Germany), S63845 (MCL-1 inhibitor) was obtained from APExBIO (Boston, MA, USA). The pan caspase inhibitor ZVAD-FMK was obtained from (Enzo Life Sciences, Bruxelles, Belgium).

### Mitochondrial copy number assay

Total DNA was isolated from >1 × 10^5^ cells using RNeasy mini kit (Qiagen, Venlo, the Netherlands). Obtained total DNA was real-time amplified in iQ SYBR Green Supermix (Bio-Rad) with the CFX connect Thermocycler (Bio-Rad). The nuclear genes GAPDH and B2M or mitochondrial genes 12S and tRNA were amplified. The obtained CT values were corrected for the corresponding calculated primer reaction efficiencies. Based on the corrected CT values, the mtDNA copy number was calculated relative to nuclear DNA copy number^27^. The primer sequences are listed in the Supplemental Table S2.

### Virus production and transduction of CD34^+^ leukemic cells or cell lines

Five lentiviral plasmids with short hairpin RNA (shRNA) targeting VMP1 were obtained from GE Healthcare Dharmacon. The shRNAs were cloned into a pLKO.1-mCherry lentiviral vector using MunI & SacII restriction enzymes (Thermo Scientific). After initial testing, two shRNAs (Clone ID TRCN0000135158 and TRCN0000138386) were selected for this study based on effective knockdown efficiency. A shRNA sequence that does not target human genes (referred to as scrambled) was used as a control. Lentiviral virions were produced by transient transfection of HEK 293 T cells with pCMV and VSV-G packing system using Polyethylenimine (Polyscience Inc. Eppelheim, Germany) or FuGENE (Promega, Leiden, the Netherlands). Retroviral virions containing pBABE-puro-mCherry-EGFP-LC3B were produced as described earlier^11^. Viral supernatants were collected and filtered through a 0.2-μm filter and subsequently concentrated using Centriprep Ultracel YM-50 centrifugal filters (Millipore, Amsterdam, The Netherlands). CD34^+^ cells were seeded in Gartners medium supplemented with cytokines (specified previously). Transduction was performed by adding 0.5 mL of ~10 times concentrated viral supernatant to 0.5 mL of medium containing 0.5 × 10^6^ cells in the presence of 4 μg/mL polybrene (Sigma-Aldrich).

### ATP assay

Luminescent ATP Detection Assay Kit (Abcam, Cambridge, UK, ab113849) was used to measure the levels of ATP, according to the manufacturer’s protocol.

### Gene ontology (GO) analysis in AML

Publicly available data of two large AML expression datasets with 460 (GSE6891^28^ and 173 (TCGA dataset^29^, samples, respectively, was analysed using the R2 Genomics Analysis and Visualization Platform (http://r2.amc.nl). Gene expression data of all genes was correlated with VMP1 expression. Correlations with a *p*-value of ≥.01 and/or with a correlation coefficient of *r* = ≤.25 were discarded. Next, genes which positively or inversely correlated with VMP1 expression in both AML datasets were compared. In total 551 (26.8% overlap) positively correlating genes and 979 (24.1% overlap) inversely correlating genes were present in both datasets. Gene ontology analysis, using David, was performed on the overlapping positively or inversely correlating genes.

### Electron microscopy

The experimental procedure for ultrastructural analysis of hematopetic cells has been described previously^30^. In brief, FACS sorted OCIM3 cells transduced with pRRL-blueberry or pRRL-VMP1-blueberry and OCIM3 cells transduced with shSCR-mCherry or shVMP1-mCherry were pelleted and subsequently fixed in 2% paraformaldehyde and 2% gluteraldehyde in 0.1 M cacodyladate buffer for 24 h at 4 °C. After fixation the cells were washed in in 0.1 M cacodyladate buffer. Cells were stained with Evans blue and subsequently embedded in low melting point agarose, as described previously^31^. Agarose pieces containing the cell pellet were dehydrated, osmicated, and embedded in Epon according to routine procedures. Semi-thin sections (0.5 mm) stained with toluidine blue were inspected using light microscopy to select for OCIM3 cells. Ultra-thin sections (60–80 nm) were cut and stained with 4% uranyl acetate in water, followed by Reynolds lead citrate. Images were taken with a Zeiss Supra55 in STEM (Oberkochen, Germany) mode with ATLAS software developed by Fibics (Ottawa, Ontario, Canada) and the CM100 (Eindhoven, the Netherlands).

### In vivo transplantations into NSG mice

Twelve- to thirteen-week-old female NSG (NOD.Cg-Prkdcscid IL2rgtm1Wjl/SzJ) mice were purchased from the Central Animal Facility breeding facility within the UMCG. Mouse experiments were performed in accordance with national and institutional guidelines and all experiments were approved by the Institutional Animal Care and Use Committee of the University of Groningen (IACUC-RuG). The experiment was performed as described previously^6^.

### Statistical analysis

An unpaired two-sided Student’s *t*-test or a Mann Whitney U test was used to calculate statistical differences. A *p*-value of <.05 was considered statistically significant.

All tables, additional material and methods sections and supplemental figure legends are available as supplemental information

## Results

### VMP1 expression is increased in a subset of CD34^+^ AML cells

**Fig. 1:**
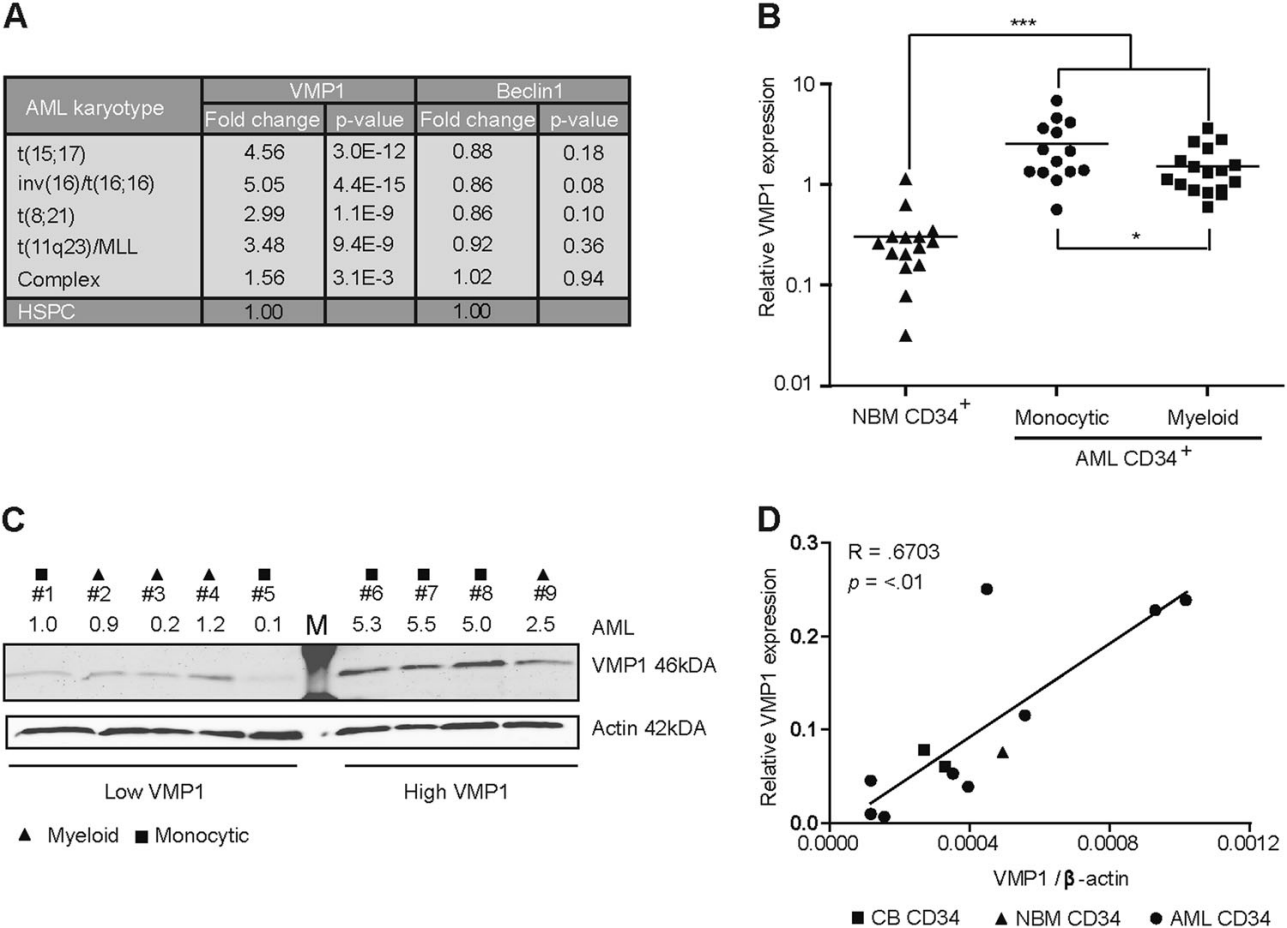
VMP1 expression is increased in a subset of AMLs. **a** Expression of VMP1 and Beclin-1 in mononuclear primary AML cells acquired from the publicly available expression dataset Bloodspot. HSPC is defined as the combined fractions of HSC, MMP, CMP, GMP and MEP. **b** Gene expression of VMP1 determined by quantitative RT-PCR in normal bone marrow (NBM) CD34^+^ cells (*n* = 15), AMLs with myeloid (*n* = 17) or monocytic characteristics (*n* = 14). **c** Western Blot showing variation in VMP1 protein levels in different AMLs (*n* = 9), β-actin was used as control and VMP1/β-actin levels are shown relative to AML1. Triangles, squares or circles indicate AMLs with a myeloid or monocytic, respectively. **d** Correlation between VMP1 mRNA levels and protein levels in primary AML CD34^+^ cells (*n* = 9, square symbols), CB CD34^+^ cells (*n* = 2, circles) and NBM CD34^+^ cells (*n* = 1, triangle). Error bars represent SD; * or *** represents *p* < .05 or *p* < .001, respectively

We previously performed transcriptome analysis on a publicly available gene expression database of normal HSPCs and AML cells (Bloodspot expression database^32^), with a focus on autophagy associated genes. This showed that several core-autophagy genes were differentially expressed in AML compared to normal HSPCs^11^. Further analysis revealed that putative autophagy protein VMP1 was expressed at significantly higher levels in AMLs compared to normal HSPCs (Fig. 1a). In contrast, the expression of the known VMP1 interaction protein Beclin-1 was not different between AML and normal HSPCs (Fig. 1a). The elevated expression of VMP1 was validated by quantitative polymerase chain reaction (qPCR), whereby the highest VMP1 expression was observed in CD34^+^ AMLs with a monocytic phenotype (Fig. 1b). Based on the VMP1 levels in Fig. 1b, AMLs with low or high VMP1 expression were selected for western blot analysis to determine VMP1 protein levels. Western blot analysis confirmed variable protein levels of VMP1 in primary AML CD34^+^ cells (*n* = 9, Fig. 1c). In addition VMP1 mRNA levels significantly correlated (*R* = 0.6703, *p* = < .01) with VMP1 protein levels (Fig. 1d, AML (*n* = 9), CB CD34^+^ cells (*n* = 2) and normal bone marrow (NBM) CD34^+^ cells (*n* = 1). Together these findings indicate that VMP1 is overexpressed in a subset of primary AML CD34^+^ cells.

### VMP1 knockdown in HSPCs results in inhibition of autophagy and an impaired in vitro expansion and in vivo engraftment

**Fig. 2:**
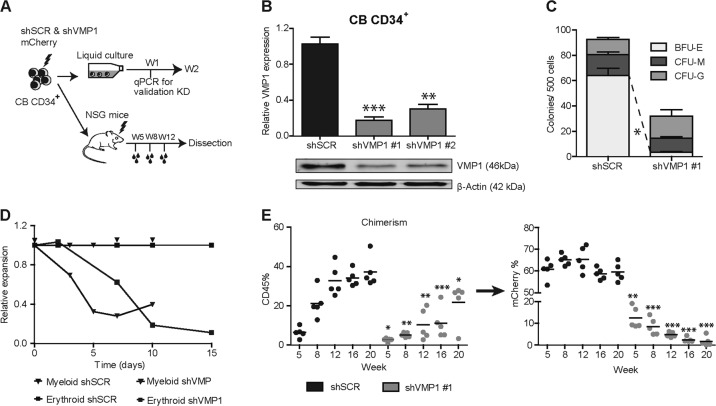
VMP1 knockdown in HSPCs results in inhibition of autophagy and an impaired in vitro expansion and in vivo engraftment. **a** Experimental scheme, shSCR-mCherry or shVMP1-mCherry transduced cord blood (CB) CD34^+^ cells were cultured in vitro under myeloid or erythroid liquid culture conditions or injected IV in sub-lethally irradiated NSG mice. Bleeds were performed at week 5, 8, and 12 after injection and analysed by FACS. **b** Knockdown efficiency of VMP1 was determined by quantitative RT–PCR and Western blotting of CB CD34^+^ cells transduced with shSCR, shVMP1 #1 or shVMP1 #2. **c** CFC assay with freshly sorted shSCR or shVMP1 #1 transduced CB CD34^+^ cells (*n* = 2). **d** Representative graph showing relative expansion of shVMP1 transduced CB CD34^+^ cells under myeloid or erythroid permissive liquid culture conditions, shSCR transduced cells were used as control (*n* = 2). **e** Percentage of engraftment represented by huCD45 percentage (left graph) and the percentage mCherry within the huCD45^+^ population (right graph). Each dot represents data from a single mouse, *n* = 5 for each group. Error bars represent SD; *, ** or *** represents *p* < .05, *p* < .01 or *p* < .001, respectively

To investigate the functional role of VMP1 in HSPCs and their progeny, human CB-derived CD34^+^ cells were transduced with lentiviral shRNAs targeting VMP1 and subsequently cultured in vitro or transplanted in vivo (Fig. 2a). A panel of five shRNAs was tested, of which two were selected based on their efficacy. Knockdown of VMP1 in CB CD34^+^ cells was confirmed at the mRNA and protein level with both shVMP1#1 and shVMP1#2 (Fig. 2b). Knockdown of VMP1 resulted in inhibition of the autophagic-flux as determined by relative accumulation of Cyto-ID after HCQ treatment at day 7 during both myeloid and erythroid liquid cultures (Supplemental Fig. S1A). As reported previously^6^, the autophagic-flux was higher under erythroid- compared to myeloid-culture conditions (Supplemental Fig. S1A). A significant reduction in erythroid progenitor (BFU-E) frequency was observed in in vitro colony assays upon knockdown of VMP1 (Fig. 2c), while no change in myeloid colony formation was observed. However, a reduction in relative expansion was observed upon knockdown of VMP1 under both erythroid and myeloid liquid culture conditions (Fig. 2d).

To assess whether VMP1 knockdown would affect the differentiation potential of CB CD34^+^ cells, the expression of myeloid (CD14, CD15) and erythroid differentiation markers (CD71, CD235A) were analysed using flow cytometry. This revealed only a minor delay in CD14 expression at day 8 (Supplemental Fig. S1B), but a stronger effect on terminal erythroid differentiation (Supplemental Fig. S1C). To study the long-term effects of VMP1 knock-down in the context of the micro-environment, transduced CD34^+^ HSPCs were cultured on MS5 bone marrow stromal co-cultures, in which VMP1 knockdown resulted in reduced expansion, as determined by the decline in percentage of mCherry positive cells (Supplemental Fig. S1D). The negative phenotype of shVMP1-transduced HSPCs was at least in part caused by increased apoptosis (Supplemental Fig. S1E), while cell cycle progression was not affected (Supplemental Fig. S1F). To assess whether VMP1 knockdown also affected in vivo engraftment, unsorted shSCR or shVMP1-mCherry-transduced CB CD34^+^ cells were transplanted into immunodeficient NSG mice (Fig. 2a). Transplanted CD34^+^ cells were ~60% mCherry-positive. Engraftment, as determined by the percentage huCD45 in peripheral blood, was significantly reduced in shVMP1 mice compared to controls (Fig. 2e, left panel). While mCherry levels for shSCR remained stable around ~60%, the contribution of the shVMP1 transduced cells to the engrafted cells over time was significantly reduced in mice with transplanted shVMP1 CD34^+^ cells, compared to controls (Fig. 2e, right panels). At sacrifice, high engraftment levels in bone marrow, spleen and liver were observed, and the contribution of shSCR-mCherry transduced cells within the CD45 compartment was around ~60% in all analyzed organs (Supplemental Fig. S1G, left panel). In contrast, the percentage of shVMP1-mCherry transduced cells within the CD45 compartment was strongly reduced (Supplemental Fig. 1G, right panel). Together these findings indicate that knockdown of VMP1 inhibits autophagic-flux and results in reduced expansion of HSPC, delayed differentiation and an impaired long-term engraftment in vivo.

### VMP1 knockdown results in inhibition of autophagy, impaired expansion, increased apoptosis and reduced cell cycle progression in leukemic cells

**Fig. 3:**
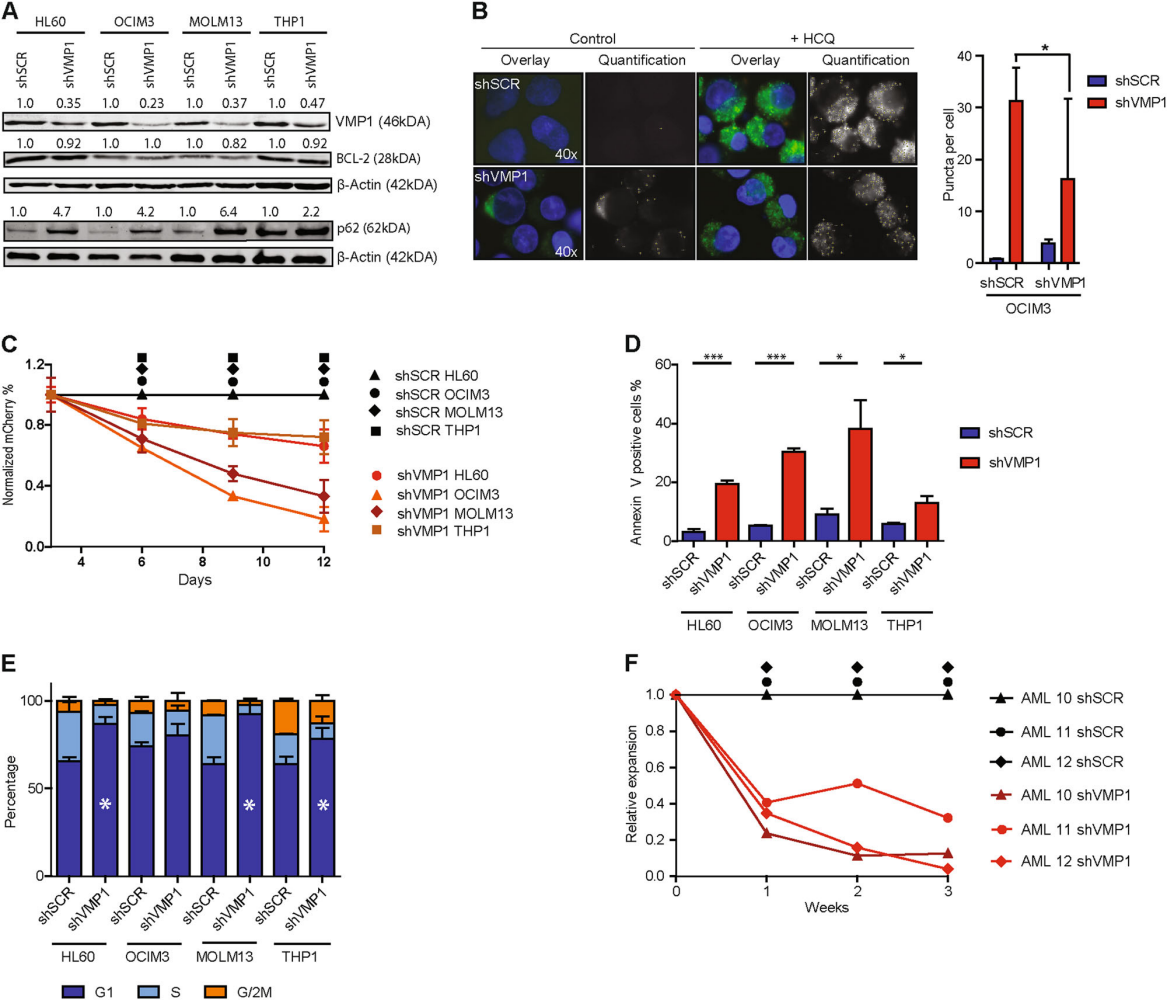
VMP1 knockdown results in inhibition of autophagy, impaired expansion, increased apoptosis and reduced cell cycle progression in leukemic cells. **a** Western blot analysis of VMP1, BCL-2, and p62/*SQSTM1* protein levels after knockdown of VMP1, β-actin was used as control. In addition, the quantification of the relative level of VMP1, BCL-2 and p62 are depicted. For each cell line, the protein levels of shVMP1 transduced cells were normalized to the protein levels in shSCR transduced control cells. **b** Left panel, representative pictures showing GFP-LC3 puncta in shSCR or shVMP1 transduced OCIM3 cells treated with or without HCQ. Dapi staining was used to count the number of cells. Right panel, quantification of GFP-LC3 puncta using ImageJ software. **c** Cell growth in time of shSCR or shVMP1 transduced leukemic cell lines; HL60, OCIM3, MOLM13 and THP1 (*n* = 3). **d** Graph showing percentage of annexin-V positive cells of shSCR or shVMP1 transduced leukemic cell lines (*n* = 3, day 6). **e** Cell cycle analysis after Hoechst staining of shSCR or shVMP1 transduced leukemic cells (*n* = 2, day 6). The * in G1-phase of shVMP1 transduced leukemic cell lines indicates a significant difference compared to shSCR control. **f** Relative expansion of AML CD34^+^ cells after knockdown of VMP1 (*n* = 3) cultured on a MS5 stromal layer. shSCR transduced AML CD34^+^ cells were used as control. Error bars represent SD; *, ** or *** represents *p* < .05, *p* < .01 or *p* < .001, respectively

Because VMP1 was shown to be differentially expressed in primary leukemic CD34^+^ cells and leukemic cell lines (Fig. 1, Supplementary Fig. 2A), the consequences of VMP1 modulation were assessed in leukemic cells. First, leukemic cell lines with previously determined autophagy activity^11^ were transduced with shVMP1 or shSCR lentivectors and knockdown efficiencies were confirmed at the protein level, while BCL-2 levels were not affected (Fig. 3a). Knockdown of VMP1 was associated with impaired autophagy, reflected by accumulation of SQSTM1/*p62* (Fig. 3a) and reduced accumulation of LC3 puncta after HCQ treatment^33^ (Fig. 3b, Supplemental Fig. S2B). The knockdown of VMP1 had a strong impact on cell growth (Fig. 3c), which was at least in part due to increased apoptosis, as determined by annexin-V positivity (Fig. 3d). The addition of the pan caspase inhibitor ZVAD-FMK partially rescued the observed phenotype (Supplemental Fig. S2C). In addition, cell cycle analysis showed that cells accumulated in G1 phase significantly (Fig. 3e). Next, AML patient-derived CD34^+^ (*n* = 3) cells were transduced with shVMP1-mCherry or shSCR-mCherry. The unsorted cells were cultured long-term in MS5 bone marrow stromal cocultures. The transduction efficiency in AML CD34^+^ cells was between 20–60% and comparable between shSCR and shVMP1 within a single AML sample (Supplemental Fig. S2D). After 4 days of culture the knockdown of VMP1 was 33 and 48% in AML10 and AML12 respectively. Similar to leukemic cell lines, knockdown of VMP1 resulted in decreased expansion of primary AML CD34^+^ cells, relative to scrambled control (Fig. 3f). Together, these results indicate that VMP1 is essential for survival and proliferation of leukemic cell lines and patient-derived AML CD34^+^ cells.

### Overexpression of VMP1 increases autophagic-flux in leukemic cells and is involved in mitochondrial turnover

**Fig. 4:**
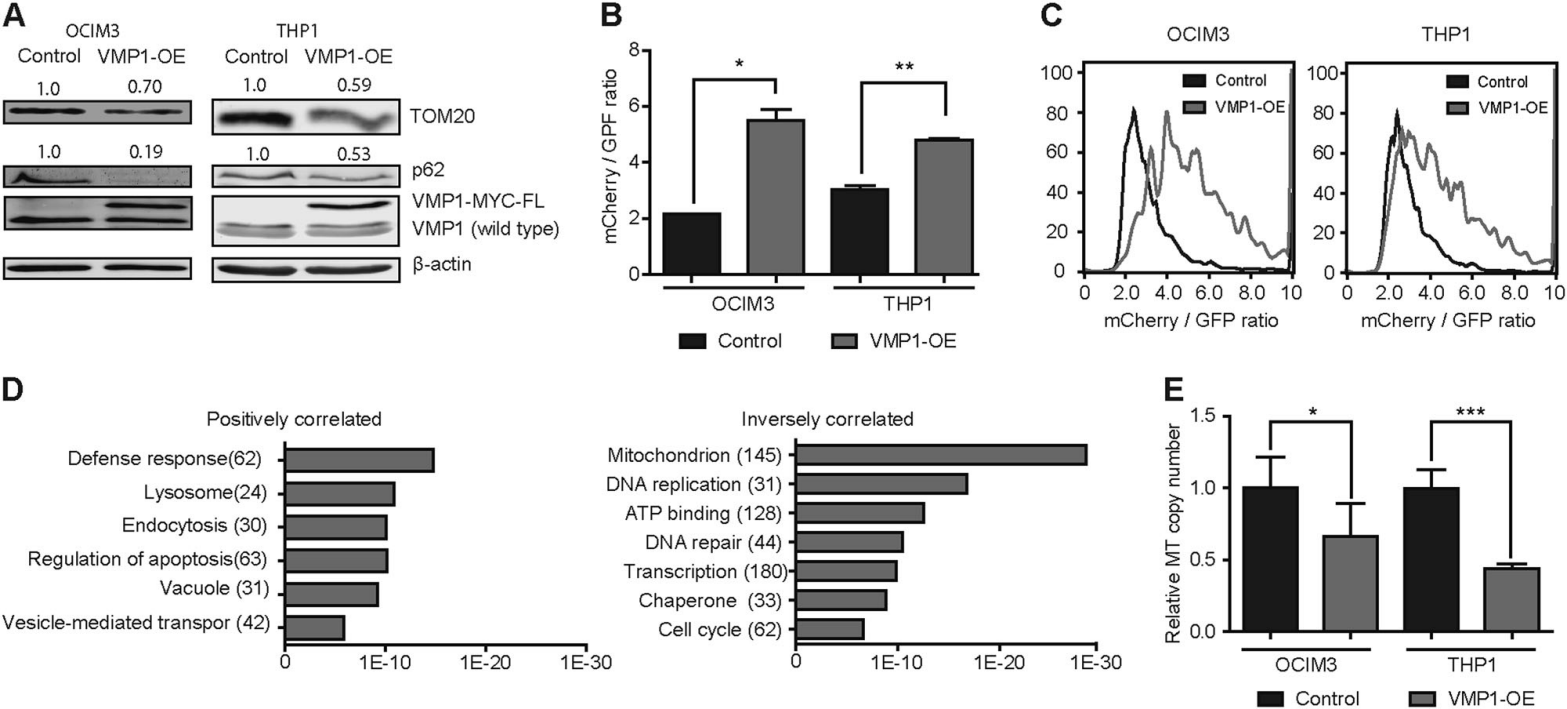
Overexpression of VMP1 increases autophagic-flux in leukemic cells and is involved in mitochondrial turnover. **a** Western blot analysis of TOM20, p62 and VMP1 in OCIM3 and THP1 cells overexpressing VMP1 (VMP1-OE) or control. **b** The mCherry/GFP ratio determined in mCherry-GFP-LC3 expressing leukemic cell lines, transduced with control or VMP1 OE. **c** Representative FACS of the mCherry/GFP ratio analysis in OCIM3 and THP1 cells. **d** Gene ontology analysis (David) was performed on two large AML patient gene expression datasets. Figures showing *p*-values of enriched gene sets, positively (left graph) or inversely (right graph) correlated with VMP1 expression in AMLs. **e** Mitochondrial copy number in OCIM3 and THP1 cells with VMP1-OE or control. Error bars represent SD; * or ** represents *p* < .05 or *p* < .01, respectively

To study the consequences of high expression of VMP1 on normal and leukemic hematopoietic cells, a lentiviral VMP1-Blueberry overexpression vector (VMP1-OE) was constructed, and overexpression of VMP1 was confirmed at the protein level (Fig. 4a). Overexpression resulted in reduced p62/SQSTM1 protein levels. (Fig. 4a). Decreased p62/SQSTM1 accumulation was confirmed by blocking autophagy at a late stage using HCQ (Supplemental Fig. S3A). In addition, overexpression of VMP1 resulted in increased relative Cyto-ID MFI, which reflects a higher LC3-dependent autophagy activity (Supplemental Fig. S3B). Next, leukemic cell lines expressing the mCherry-GFP-LC3 autophagy reporter were transduced with control or VMP1-OE cells to assess the impact on the autophagy flux. An increased mCherry/GFP ratio was observed in OCIM3 and THP1 cells overexpressing VMP1, confirming that the LC3-dependent autophagy-flux was increased (Fig. 4b–c). The increased autophagy did not affect the cell proliferation of the cell lines.

To obtain more insight into the role of VMP1 in AML, two large AML expression datasets^28,29^ were analysed using the R2 Genomics Analysis and Visualization Platform. Gene ontology analyses (GO) was performed on genes correlating with VMP1 expression. GO analysis revealed that positively correlated genes were enriched for the GO terms lysosome, vacuole and vesicle transport, while inversely correlated genes were enriched for the GO terms mitochondrion, DNA replication, ATP binding, apoptosis and cell cycle (Fig. 4d). Because high VMP1 expression in AML cells is inversely correlated with gene signatures associated with mitochondria (Fig. 4d, right panel), mitochondrial function was assessed after modulation of VMP1 expression. Overexpression of VMP1 led to a significant decrease in mitochondrial DNA (mtDNA) copy number relative to nuclear DNA (nucDNA) in OCIM3 and THP1 cells (Fig. 4e). In line with these results, TOM20 which is a marker for mitochondrial mass, was decreased after overexpression of VMP1 (Fig. 4a). Conversely, shVMP1 transduced leukemic cells had an increase in mtDNA copy number in cell lines. (Supplemental Fig. S3C). However, this increase was not observed in MOLM13 cells. Together these findings indicate that overexpression of VMP1 is involved in mitochondrial turnover.

### Ultrastructural analysis and mitochondrial function in OCIM3 cells after VMP1 modulation

**Fig. 5:**
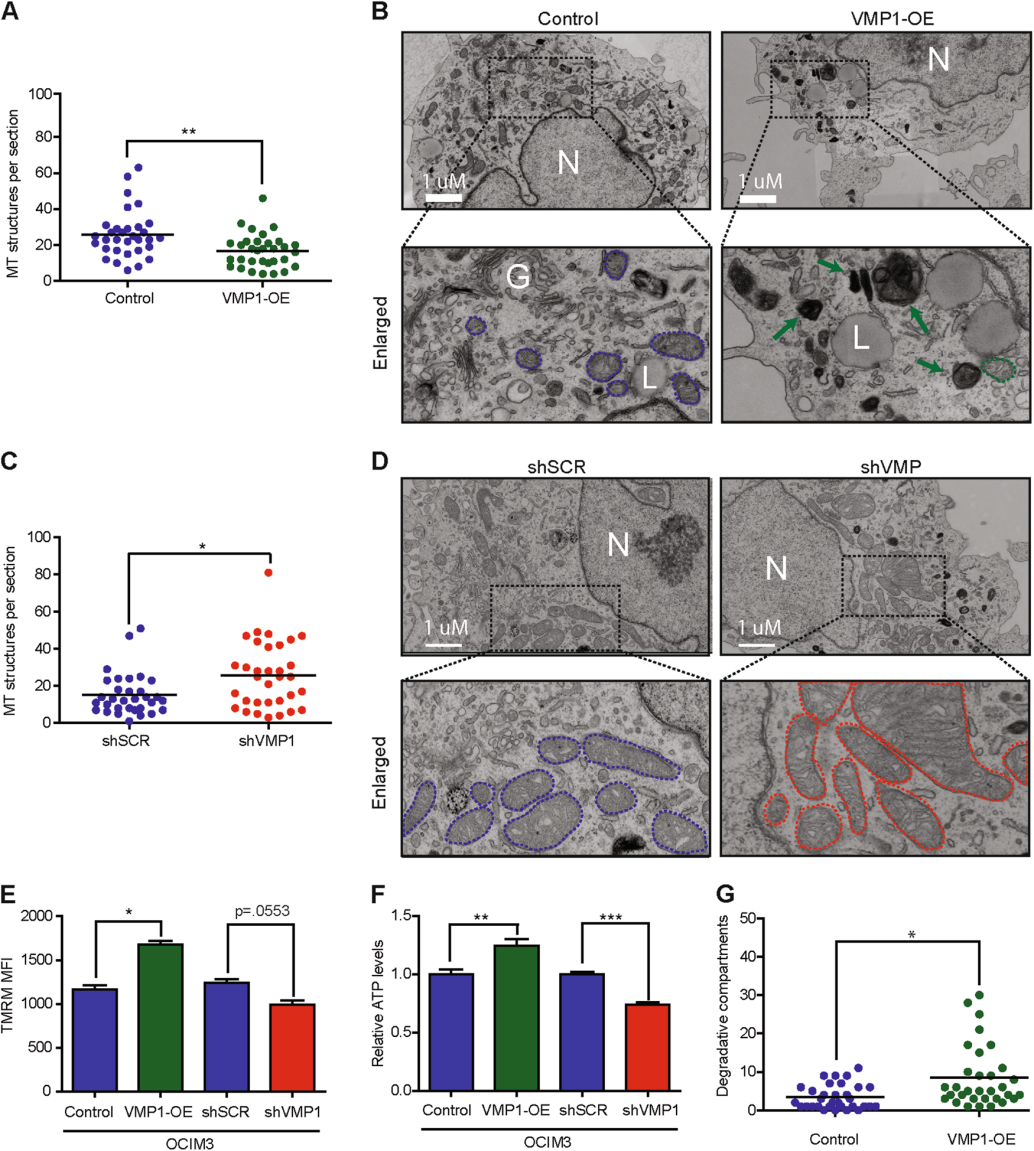
Ultrastructural analysis and mitochondrial function in OCIM3 cells after VMP1 modulation. **a** Quantification of *mitochondrial structures per section* in OCIM3 cells transduced with lentiviral vectors for overexpression of VMP1 (VMP1-OE) or control (*n* > 32 sections per group). **b** Representative ultrastructural pictures of OCI3M cells overexpression VMP1 or control, N = nucleus, L = lipid droplet, G = Golgi, green arrow = whorls/degradative compartments. The blue (control) or green (VMP1-OE) dotted lines indicate mitochondrial structures. **c** Quantification of *mitochondrial structures per section* in OCIM3 cells with knockdown of VMP1 (shVMP1) or control vectors (*n* > 32 sections per group). **d** Representative ultrastructural pictures of OCI3M cells with knockdown of VMP1 or control. N = nucleus. The blue (control) or red (shVMP1) dotted lines indicate mitochondrial structures. **e** FACS analysis of mitochondrial membrane potential (MMP) after tetramethylrhodamine (TMRM) staining in OCIM3 with VMP1 overexpression, VMP1 knockdown or control (*n* = 3). **f** ATP levels measured in OCIM3 with VMP1 overexpression, VMP1 knockdown or control (*n* = 4). **g** Electron microscopy, quantification of onion-like multilamellar membrane structures called degradative compartments per section of OCIM3 cells, transduced with VMP-OE or control (*n* ≤ 35 cells per group). Examples of degradative compartments are indicated by green arrows in **b** right panels. Error bars represent SD; * or ** represents *p* < .05 or *p* < .01, respectively

To obtain more insight into the involvement of VMP1 in mitochondrial quality control, we analyzed OCIM3 cells ultrastructurally with electron microscopy (EM) after lentiviral overexpression or knockdown of VMP1. OCIM3 cells overexpressing VMP1 displayed a reduction in the number of mitochondrial structures compared to control cells (Fig. 5a, *p* = < .01). Representative ultrastructural images are shown in Fig. 5b. In the shVMP1-transduced OCIM3 cells mitochondria were on average ~24% larger and swollen compared to control (Supplemental Fig. 4A). Moreover, in contrast to VMP1-OE, cells with knockdown of VMP1 had elevated numbers of mitochondrial structures (Fig. 5c–d, *p* = < .05). In addition, the mitochondrial function was analyzed after VMP1 modulation. The mitochondrial membrane potential (MMP) measured by tetramethylrhodamine (TMRM) was significantly increased in OCIM3 cells overexpressing VMP1, while there was a trend for reduced MMP upon VMP1 knockdown (Fig. 5e). Although the number of mitochondria in VMP1 overexpressing cells was reduced (Fig. 4), these cells had higher levels of ATP content (Fig. 5f). Conversely, knockdown of VMP1 resulted in decreased ATP production with concomitant increased ROS levels, indicating a loss in mitochondrial function (Fig. 5f, Supplemental Fig. S4B–C). In line with these results, increased ROS levels were also observed with the MitoSOX tracer confirming that ROS is of mitochondria origin (Supplementary Fig. S4, D). Additional analysis by EM revealed that cells overexpressing VMP1 had an increased number of onion-like multilamellar membrane structures, also called whorls (Fig. 5a, g, 1.5 fold, *p* = < .05). These structures are associated with lysosomal mediated degradation of intra-cellular parts, also termed the degradative compartment^34^, and are associated with an increased autophagy flux. Together, the increase in autophagy, the reduced number of mitochondria structures in response to VMP1 overexpression are indicative of increased turnover of mitochondria.

### Overexpression of VMP1 interferes with venetoclax induced apoptosis

**Fig. 6:**
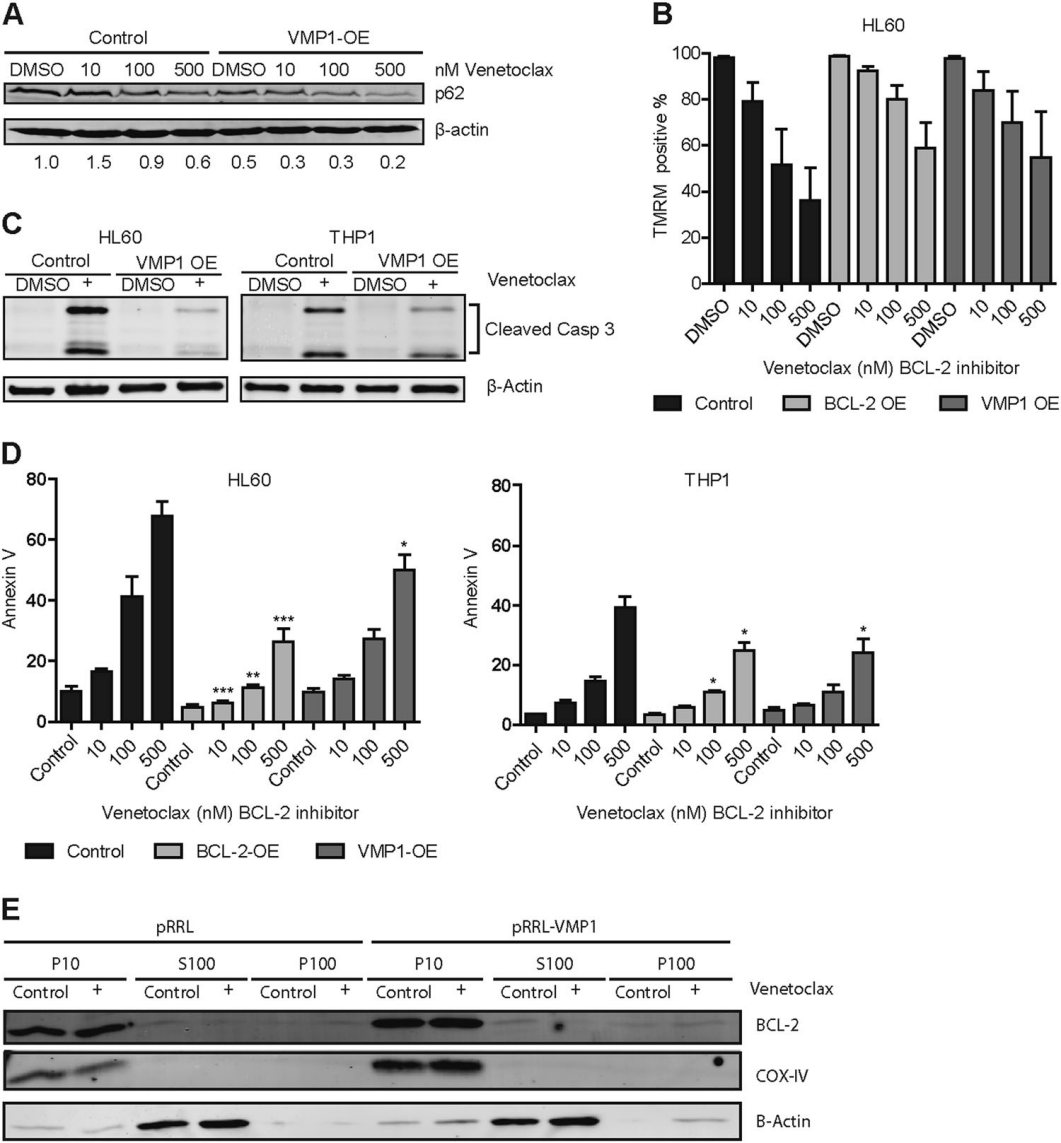
Overexpression of VMP1 interferes with venetoclax induced apoptosis. **a** Western blot of p62 in THP1 cells overexpressing VMP1 or control treated with different concentrations of venetoclax, β-actin was used as control. (*n* = 2) **b** Bar graph showing the percentage of TMRM positive cells. HL60 cells overexpressing VMP1, BCL-2 or control were incubated for 24 h with different concentrations of venetoclax. (*n* = 3) **c** Western Blot showing cleaved caspase 3 in HL60 and THP1 cells overexpressing VMP1 or control after 24 h incubation with 25 nM venetoclax (*n* = 2). **d** Leukemic cell lines with lentiviral overexpression of VMP1, BCL-2 or control were treated 24 h with venetoclax and apoptosis was measured by annexin-V staining with FACS (*n* = 4). **e** Western blot showing BCL-2, COX-IV, and β-Actin in the mitochondrial (P10), cytosolic (S100) or endoplasmic reticulum (P100) fractions of HL60 cells overexpressing VMP1 or control, treated with or without venetoclax. Error bars represent SD; *, ** or *** represents *p* < .05, *p* < .01 or *p* < .001, respectively

BCL-2 protein family members regulate apoptosis by controlling the permeability of mitochondria^35^. Interestingly, VMP1 has been shown to contain a BH3-binding domain, which is an important characteristic of the BCL-2 protein family^36^. The specific BCL-2 inhibitor venetoclax has been shown to disrupt the BH3 dependent BCL-2/Beclin-1 interaction, thereby activating autophagy^37,38^. First, we studied the consequences for autophagy activity after venetoclax treatment in the context of high VMP1 expression. As expected, in THP1 cells p62 levels declined in a dose-dependent manner with increasing concentration of venetoclax, which is indicative for increased autophagic-flux (Fig. 6a). Basal p62 levels were reduced in THP1 cells overexpressing VMP1, while p62 levels further declined with increasing concentrations of venetoclax (Fig. 6a). Next, we evaluated the effect of high VMP1 expression on the threshold for mitochondrial outer membrane permeabilization (MOMP). The initiation of MOMP is preceded by loss of mitochondrial membrane potential (MMP) and results in caspase-dependent apoptosis^35^. Leukemic cells overexpressing either BCL-2 or VMP1 were treated with increasing concentrations of venetoclax and the MMP was determined after tetramethylrhodamine (TMRM) staining in the context of BCL-2 and VMP1 overexpression. Venetoclax-induced loss of MMP could be partially rescued by VMP1 or BCL-2 overexpression (Fig. 6b and Supplemental Fig. 5A). In addition, venetoclax induced apoptotic response in HL60 and THP1 cells, as determined by caspase-3 cleavage and annexin-V staining, could partially be rescued by overexpressing BCL-2 or VMP1 (Fig. 6c–d and Supplemental Fig. S5A). Inhibition of autophagy with HCQ did not reverse the VMP1-medated rescue of venetoclax cytotoxicity (Supplementary Fig. 5B). Next, we validated whether inhibition of autophagy by knocking down VMP1 or the essential autophagy gene ATG7 would affect venetoclax mediated cell death. In contrast to overexpression of VMP1, knockdown of VMP1 or ATG7 in combination with venetoclax treatment resulted in enhanced cell death, although no synergistic effects were observed with the combination (shVMP1 31 ± 3.7% vs. shVMP1 plus venetoclax 53 ± 3.7% reduction in survival *p* = < 0.05 and shATG7 18% ± 2.8% vs. shATG7 plus venetoclax 45 ± 2.5% reduction in survival *p* = < 0.01).

To study the specificity of effects, leukemic cell lines were treated with the specific and potent MCL-1 inhibitor S63845^39^. In contrast to BCL-2 overexpression, VMP1 overexpression did not rescue S63845-mediated apoptosis (Supplemental Fig. S5C). Finally we tested if high VMP1 expression would affect BCL-2 expression or cellular localization. BCL-2 protein levels did not change after treatment with increasing concentrations of venetoclax in VMP1 overexpressing cells (Supplemental Fig. S5D). Next, cellular fractionation was performed of HL60 cells overexpressing VMP1 or control vector. From total cell lysates the mitochondrial (P10), cytosolic S100 or endoplasmic reticulum fraction (P100) were purified. Purification of the mitochondrial fraction and cytosolic fraction was confirmed by enrichment for COX-IV or β-actin, respectively. Interestingly, under all tested conditions BCL-2 was primarily present in the mitochondrial fraction, suggesting that relocalization of BCL-2 did not cause the observed resistance against venetoclax-mediated cell death in high VMP1 expressing cells (Fig. 6e).

Together, these data indicate that overexpression of BCL-2 and VMP1 increase the threshold for venetoclax-mediated loss of MOMP, thereby reducing apoptosis-mediated cell death.

## Discussion

The present study indicates that VMP1 is involved in autophagy and mitochondrial turn-over in normal hematopoietic and leukemic cells. Blocking VMP1 expression impairs the autophagy flux, coinciding with reduced proliferation and survival of normal and leukemic HSPC and impaired in vivo engraftment. Previous studies on VMP1 function have been done primarily in solid tumors^40,41^, but gene expression studies in hematopoietic cells showed that VMP1 is differentially expressed in hematopoietic cell lineages, including HSPCs. In the leukemic counterpart, VMP1 can be overexpressed in all AML subcategories independent of the molecular and genetic makeup. Functional studies have shown that VMP1 disrupts the binding of BCL-2 to Beclin-1 and consequently de-represses autophagy^23^. These findings are in line with our results showing that reduced VMP1 expression resulted in inhibition of LC3-dependent autophagy, while overexpression enhanced LC3-dependent autophagy-flux. This would also suggest that AMLs with high VMP1 expression are primed for robust autophagy activation in response to cellular stress. In addition, our study showed that high VMP1 expression is inversely correlated with genes enriched for the GO-terms associated with mitochondria, which is in line with studies in Hela cells showing that VMP1 co-localizes with mitochondrial structures^34,37^. Based on functional studies and electron microscopy, our results suggest that VMP1 regulates mitochondria quantity and quality by affecting mitophagy. Higher VMP1 expression reduces mitochondrial copy number and TOM20 expression and increases mitochondrial membrane potential and ATP production. This is indicative for improvement in quality of the remaining mitochondria, while reduced VMP1 expression generated the opposite results. In addition, knockdown of VMP1 resulted in swollen mitochondria and increased ROS levels, which is most likely the consequence of accumulating (dysfunction) mitochondria, resulting in cell death which coincides with the loss of mitochondrial membrane potential and ATP levels. Previous studies with ATG12, ATG5 or ATG7 knockout mice have reported comparable results: accumulating dysfunctional mitochondria and ROS^5,42–44^.

The higher expression of VMP1 in AML CD34^+^ cells might be protective for AML cells in the hypoxic bone marrow micro-environment, where control of mitochondria content and ROS production by autophagy is crucial for maintaining an immature phenotype^5^. Similar to other pro-autophagy genes^45^, VMP1 expression can be upregulated under hypoxia^46^. However, in leukemic cell lines TOM20 and VMP1 protein levels both declined when the cells were cultured under hypoxia (data not shown). Therefore it is more likely that the mitochondrial turnover during hypoxia is controlled by BNIP3 and BNIP3-L, which demonstrate a strong upregulation in response to hypoxia exposure^45^.

The threshold for MOMP, and consequently for cytochrome-c release-dependent caspase activation, is regulated by the expression level of BCL-2 protein family members, which might account for drug resistance in a subgroup of AML patients^47–50^. Recently, promising results have been obtained with venetoclax in relapsing AML patients in conjunction with low-dose chemotherapy^51^. Predictive markers have been identified for reduced sensitivity for venetoclax, such as increased expression of BCL-XL or MCL-1^39,47,52,53^. The present study indicates that VMP1 overexpression is an additional predictive maker for resistance against venetoclax in AML. VMP1 overexpression resulted in an increase in MMP, which could in turn increase the threshold for venetoclax-mediated apoptosis. Mitochondria play a central role in regulation of apoptosis^54^. Cells with more mitochondrial content where shown to be more prone to undergo apoptosis^55^. Therefore, VMP1 overexpression could potentially inhibit pro-apoptotic signalling by increased turnover of dysfunctional mitochondria. However, these protective effects of VMP1 are only partially regulated by autophagy induction since HCQ did not reverse the VMP1-mediated rescue of venetoclax induced apoptosis.

In summary, the results demonstrate that VMP1 is essential for HSPC and AML cells and is involved in mitochondrial quality control. High VMP1 expression is protective against loss of membrane potential and apoptosis induced by venetoclax.

## Supplementary information

Supplemental information

Supplemental Figure S1

Supplemental Figure S2

Supplemental Figure S3

Supplemental Figure S4

Supplemental Figure S5
