# Supplementary material for: Elevated VMP1 expression in acute myeloid leukemia amplifies autophagy and is protective against venetoclax-induced apoptosis
Source: Cell Death Dis. 2019 May 29;10(6):421. doi: 10.1038/s41419-019-1648-4 (PMC6541608; doi:10.1038/s41419-019-1648-4)
Supplement: Supplementary file 1 — Supplemental information [file 41419_2019_1648_MOESM1_ESM.docx]

**Supplemental figure legends**

**Supplemental Figure S1 VMP1 knockdown results impaired expansion and increased apoptosis in leukemic cells.**

**A)** Relative Cyto-ID MFI in shSCR and shVMP1 transduced cord blood (CB) CD34^+^ cultured under erythroid or myeloid permissive conditions and treated overnight with or without HCQ. **B-C)** Representative experiment of myeloid differentiation markers CD14 and CD15 and erythroid differentiation makers CD71 and GPA in time of shSCR and shVMP1 transduced CB CD34^+^ cells of two independent experiments. **D)** Representative graph showing the percentage of mCherry positive cells in an MS5 coculture of unsorted CD34^+^ cells, transduced with shSCR-mCHerry or shVMP1-mCHerry (n=2). **E-F)** Annexin-V percentage and cell cycle distribution of shSCR or shVMP1 transduced CB CD34^+^ cells at day 4 and 6 after transduction (n-3). **G)** Engraftment (percentage huCD45) at time of sacrifice in bone marrow, spleen and liver (left graph) and the mCherry percentage within the huCD45^+^ population (right graph). Error bars represent SD; *, ** or *** represents p<.05, p<.01 or p<.001, respectively.

**Supplemental Figure S2:** **VMP1 knockdown results impaired expansion and increased apoptosis in leukemic cells**

**A)*.*** VMP1 protein levels corrected for β-Actin in different leukemic cell lines. **B)**. Left panel, representative pictures showing GFP-LC3 puncta in shSCR or shVMP1 transduced MOLM13 cells treated with or without HCQ. Right panel, quantification of LC3 puncta. **C)** THP1 cells transduced with shSCR or shVMP1 were treated with or without 20 µM ZVAD-FMK (pan caspase inhibitor). Normalized cell expansion was shown at day 6 of culture relative to untreated shSCR transduced control cells. **D)** Representative FACS plots of shSCR-mCherry or shVMP1-mCherry transduced primary AML CD34^+^ cells at day 3 and after 2 weeks.

**Supplemental Figure S3:** **Overexpression of VMP1 increases autophagic flux in leukemic cells and is involved in mitochondrial turnover**

**A)** Western blot showing p62 accumulation after overexpression of VMP1 or control vector in HL60 and THP1 cells treated with or without HCQ. β-actin was used as control **B)** HL60 and THP1 cells transduced with VMP1 or control vector were treated with or without HCQ. Bar graph showing the relative increase in Cyto-ID MFI after inhibition of autophagy with HCQ. **C)** Mitochondrial copy number in leukemic cell lines transduced with shSCR or shVMP1 after 3 days of knockdown.

**Supplemental Figure S4:** **Ultrastructural analysis of OCIM3 cells after VMP1 modulation**

**A**) Electron microscopy analysis of total mitochondrial surface area in OCIM3 cells transduced with shSCR or shVMP1 (n=324 mitochondria per group). **B)** ATP levels, measured in leukemic cell lines transduced with shSCR or shVMP1. **C)** FACS analysis of CellROX (ROS) MFI in leukemic cell lines cells transduced with shSCR or shVMP1. **D)** Mitosox MFI in OCIM3 and MOLM13 cells transduced with shSCR or shVMP1. Error bars represent SD; *, ** or *** represents p<.05, p<.01 or p<.001, respectively.

**Supplemental Figure S5:** **Overexpression of VMP1 interferes with venetoclax induced apoptosis in leukemic cells**

**A)** HL60 cells overexpressing BCL-2, VMP1 or control were incubated for 24 hrs with different concentrations of venetoclax. Representative FACS plots show the percentage of TMRM or annexin-V positive cells. **B)** HL60 and THP1 cells overexpressing VMP1 or control vector were treated with Venetoclax, HCQ or a combination. Bar graphs showing the percentage of viability after 16 hour treatment. **C)** Leukemic cell lines with lentiviral overexpression of VMP1, BCL-2 or control were treated 24 hrs with MCL-1 inhibitor S63845 and apoptosis was measured after annexin-V staining with FACS (n=4). **D)** Western Blot showing BCL-2, VMP1 and β-Actin in HL60 cells overexpressing VMP1 or control vector, treated with different concentrations venetoclax.

**Supplemental tables**

| **Supplemental Table S1** Characteristics of AML patients | | | | | |
| --- | --- | --- | --- | --- | --- |
| **#** | **Age** | **Sex** | **CD34%** | **Defined molecular mutations** | **Cytogenetics** |
| 1 | 61 | F | 61 | FLT3-ITD | t(11;20) |
| 2 | 69 | M | 74 | TP53 | t(3;5) and -5 |
| 3 | 64 | M | 74 | n.a. | NK |
| 4 | 52 | M | 52 | FLT3-ITD, | NK |
| 5 | 77 | F | 70 | FLT3-ITD | NK |
| 6 | 59 | F | 59 | n.a. | Inv(16) |
| 7 | 75 | M | 76 | FLT3-ITD | NK |
| 8 | 43 | F | 28 | FLT3-ITD | NK |
| 9 | 60 | M | 39 | FLT3-ITD | t(3;5) and +8 |
| 10 | 63 | M | 40 | TET2, EZH2, FLT3-ITD, RUNX1, ASXL1, CEBP | +8 |
| 11 | 67 | F | 55 | n.a. | inv(16) |
| 12 | 74 | M | 72 | FLT3-ITD | -2 and der(14)t(2;14) |

**Supplemental table S1**

*Patient characteristics****.*** *Abbreviations:* *M, male; F, female; n.a., not available; NK, normal* karyotype; *TP53, Tumor protein 53; IDH1, isocitrate dehydrogenase 1; IDH2, Isocitrate dehydrogenase 2; FLT3-ITD, Fms-like tyrosine kinase 3 internal tandem duplication; DNMT3A, DNA Cytosine-5-Methyltransferase 3 Alpha; RUNX1, Runt-related transcription factor 1; ASXL1, Additional Sex Combs Like 1; CEBP, CCAAT Enhancer Binding Protein; TET2, Tet Methylcytosine Dioxygenase 2; EZH2, Enhancer Of Zeste 2 Polycomb Repressive Complex 2 Subunit.* ^1, 2^

| **Supplemental Table S2.** Primers mitochondrial copy number assay | | |
| --- | --- | --- |
| **Gene** | **Forward primer** | **Reverse primer** |
| **B2M** | 5’- TGCTGTCTCCATGTTTGATGTATCT -3’ | 5’- TCTCTGCTCCCCACCTCTAAGT -3’ |
| **GAPDH** | 5’- TACTGGTGTCTTCACCACCA -3’ | 5’- CAGGATGCATTGCTGACAATC -3’ |
| **12sRNA** | 5’- AGAACACTACGAGCCACAGC -3’ | 5’- ACTTGCGCTTACTTTGTAGCC -3’ |
| **tRNA-Leu** | 5’- CACCCAAGAACAGGGTTTGT -3’ | 5’- TGGCCATGGGTATGTTGTTA -3’ |

| **Supplemental Table S3:** Antibodies used for flowcytometry analysis | | | |
| --- | --- | --- | --- |
| **Antibody** | **Fluorochrome** | **Clone number** | **Company** |
| Anti-CD34 | Pe-Cy7 | 8G12 | BD Pharmingen |
| Anti-CD45 | BV421 | HI30 | BioLegend |
| Anti-CD14 | PE | HCD14 | BioLegend |
| Anti-CD15 | PerCP | W6D3 | BioLegend |
| anti-CD71 | Alexa700 | MEM-75 | EXBIO |
| Anti-CD235A (GPA) | FITC | GA-R2 (HIR2) | BD Pharmingen |

| **Supplemental Table S4.** Primers quantitative PCR | | |
| --- | --- | --- |
| **Gene** | **Forward primer** | **Reverse primer** |
| **RPS11** | 5’-AAGATGGCGGACATTCAGAC-3’ | 5’-AGCTTCTCCTTGCCAGTTTC-3’ |
| **RPL27** | 5’-TCCGGACGCAAAGCTGTCATCG-3’ | 5’-TCTTGCCCATGGCAGCTGTCAC-3’ |
| **VMP1** | 5’-CAGATGAAGAGGGCACTGAAGG-3’ | 5’-CTCCGATTGCTGTACCGATACC-3’ |

**Supplemental material & methods**

**Western blotting**

Western blot analysis was performed using standard techniques. In brief, cells were lysed in Laemmli buffer and boiled for 5 min. Equal amounts of total lysate were analyzed by SDS-polyacrilamide gel electrophoresis. Proteins were transferred to polyvinylidene difluoride (PVDF) membrane (Millipore, Amsterdam, the Netherlands) by semidry electroblotting. Membranes were blocked in Odyssey blocking buffer (Westburg, Leusden, the Netherlands) prior to incubation with the appropriate antibodies according to the manufacturer’s conditions. Membranes were washed, incubated with secondary antibodies labelled with alexa680 or IRDye800 (Invitrogen, Breda, the Netherlands) and developed by Odyssey infrared scanner (Li-Cor Biosciences, Lincoln, NE, USA).

**Flow cytometry analysis and apoptosis measurements**

After isolation, cells were resuspended in PBS and subsequently incubated for 30 min at 4°C with anti-human CD34, CD45, CD235A/GPA, CD71, CD14 or CD15. All used antibodies are indicated in supplemental Table S3. After incubation, cells were washed and optionally incubated for 30 min at 37˚C using Cyto-ID Autophagy Detection dye (ENZ-51031-0050, Enzo Life Science, Bruxelles, Belgium). The cells were subsequently washed and analysed by flow cytometric analysis (FACS). Apoptosis was quantified by staining with APC-conjugated Annexin-V (Beckton Dickinson, Franklin Lakes, USA) according to manufacturer’s protocol. Mitochondrial membrane potential was determined using TMRM, according to the manufactures protocol. Mitochondrial ROS was measured with MitoSOX and analysed by FACS (Thermofisher, Landsmeer, the Netherlands). Cell cycle analysis was performed by staining cells in 5mg/ml Hoechst 33342 (Invitrogen) at 37°C for 45 minutes. Cells were subsequently washed and measured in the presence of Hoechst 33342. All data was analyzed using FlowJo (Tree Star, Oregon, USA) software.

**Quantitative real-time PCR**

Quantitative RT-PCR was performed to analyse the mRNA levels of VMP1 or BCL-2. Total RNA was isolated from at least 1x10^5^ cells using the RNeasy kit (Qiagen, Venlo, the Netherlands). RNA was reverse transcribed with iScript reverse Transcription kit (Bio-rad Veenendaal, the Netherlands). Obtained cDNA was real-time amplified in iQ SYBR Green Supermix (Bio-Rad) with the CFX connect Thermocycler (Bio-Rad). RPL27 and RPS11 were used as housekeeping genes. The primer sequences are listed in the Supplemental Table S4.

**Cellular fractionation**

The cellular fractionation was adapted from Vander Heiden MG *et al^3^*. Briefly, 10 × 10^6^ cells per sample were washed twice with ice-cold phosphate-buffered saline (PBS) then kept on ice for 20 minutes in 800 µL buffer A, containing 20 mM HEPES-KOH (pH 7.2), 100 mM KCl, 1.5 mM MgCl_2_, 1 mM EDTA, 1 mM EGTA, 250 mM sucrose, DTT and protease inhibitors. The cell suspension was gently homogenized with a Dounce homogenizer (20 strokes). The homogenate was centrifuged at 750g for 10 minutes, at 4^o^C (to remove un-lysed cells and nuclei), and the supernatant subjected to further centrifugation at 10 000g for 25 minutes at 4^o^C with a optima Max-1 ultracentrifuge (Beckman)**.** This pellet, containing mitochondria was washed with 500 µL buffer A, was designated P10. The supernatant was subjected to further ultracentrifugation at 100 000g for 45 minutes**,** 4^o^C**.** The resulting pellet and supernatant, representing the endoplasmic reticulum and cytosolic fractions, were designated as P100 and S100, respectively. After fractionation the volumes of the P10 and P100 fractions were adjusted to the same volume as the S100 fraction. The purity of the cellular fractions was examined by Western blotting using β-actin and COX-IV, as cytoplasmic and mitochondrial markers, respectively.

**References:**

1. Vardiman, J.W. et al. Introduction and overview of the classification of the myeloid neoplasms. In WHO Classification of Tumors of Haematopoietic and Lymphoid Tissues (eds Swerdlow, S. H.) (IARC, Lyon, France, 2008).

2. Swerdlow SH et al. WHO Classification of Tumours of Haematopoietic and Lymphoid Tissues WHO Classification of Tumours, Revised 4th Edition, Volume 2, 2017.

3. Vander Heiden, M.G., Chandel, N.S., Williamson, E.K., Schumacker, P.T., Thompson, C.B. Bcl-xL regulates the membrane potential and volume homeostasis of mitochondria. *Cell* 1997, **91**(5)**:** 627-637.
