# Supplementary figures and images for: Elevated VMP1 expression in acute myeloid leukemia amplifies autophagy and is protective against venetoclax-induced apoptosis
Source: Cell Death Dis. 2019 May 29;10(6):421. doi: 10.1038/s41419-019-1648-4 (PMC6541608; doi:10.1038/s41419-019-1648-4)

Supplemental Figure S1.

A

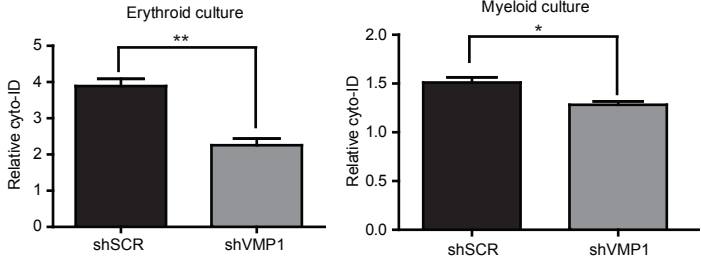

B

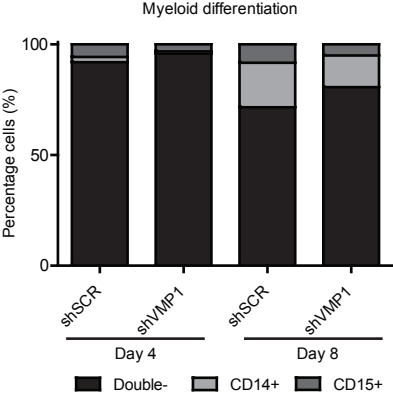

C

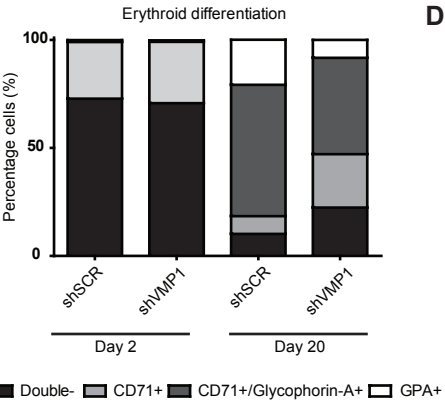

D

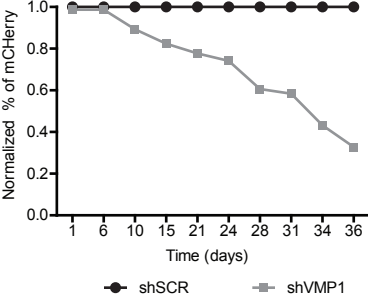

E

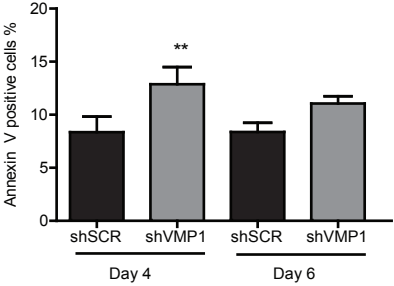

F

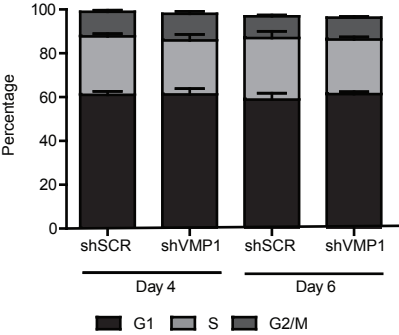

G

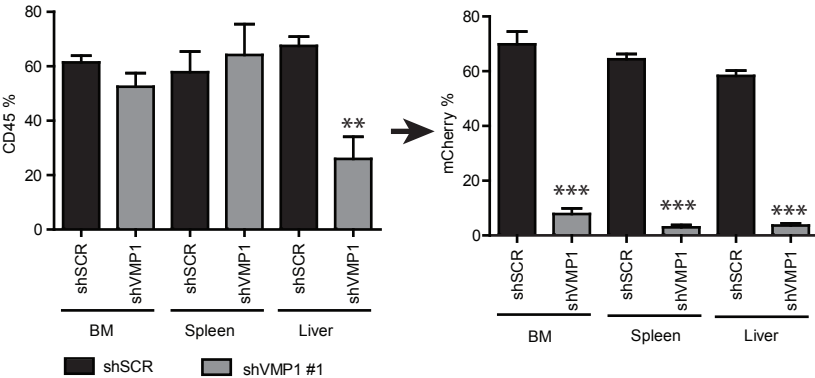

Supplement: Supplementary file 2 — Supplemental Figure S1 [file 41419_2019_1648_MOESM2_ESM.pdf]

# Supplemental Figure S2.

**A**

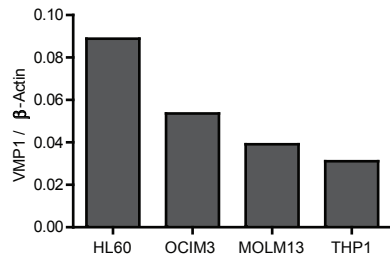

**B**

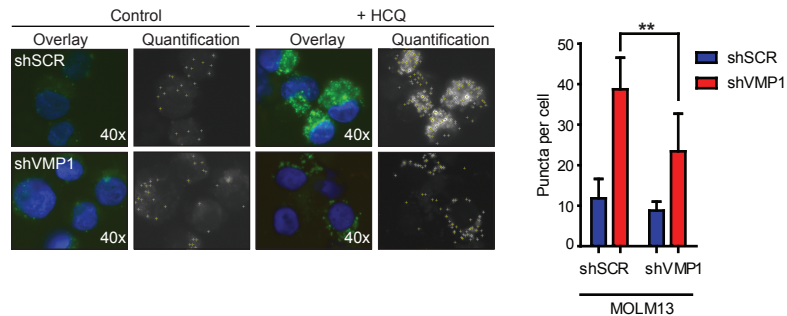

**C**

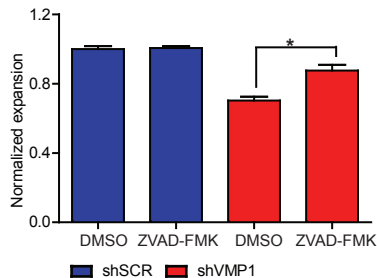

**D**

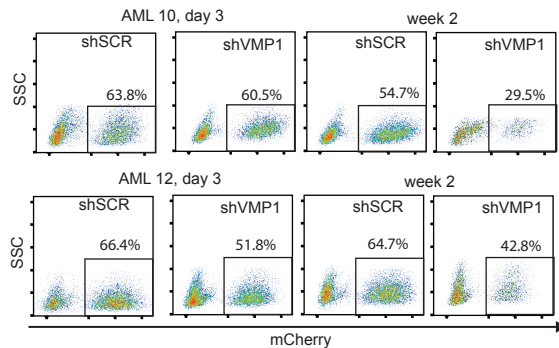

Supplement: Supplementary file 3 — Supplemental Figure S2 [file 41419_2019_1648_MOESM3_ESM.pdf]

**Supplemental Figure S3.**

**A**

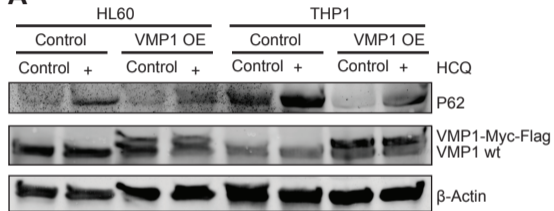

**B**

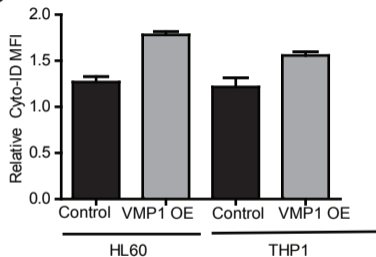

**C**

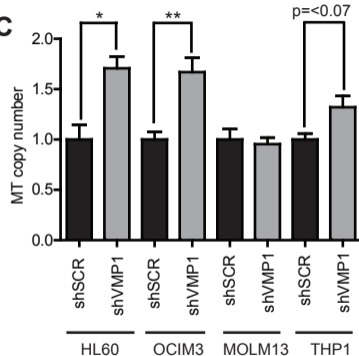

Supplement: Supplementary file 4 — Supplemental Figure S3 [file 41419_2019_1648_MOESM4_ESM.pdf]

**Supplemental Figure S4.**

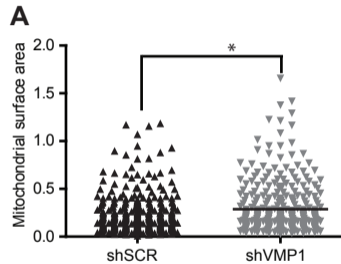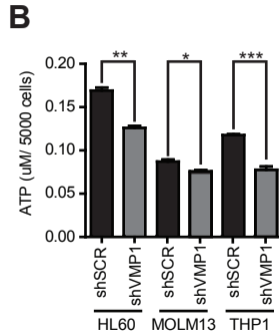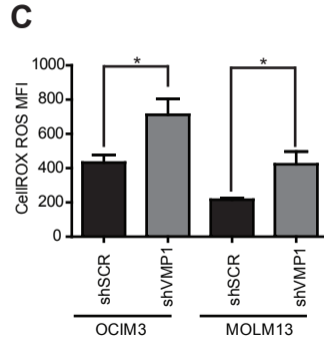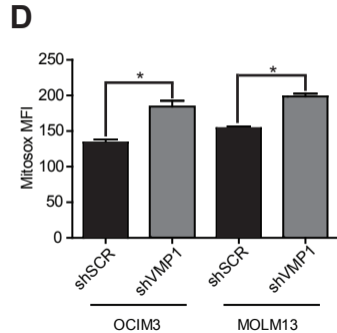

Supplement: Supplementary file 5 — Supplemental Figure S4 [file 41419_2019_1648_MOESM5_ESM.pdf]

Supplemental Figure S5.

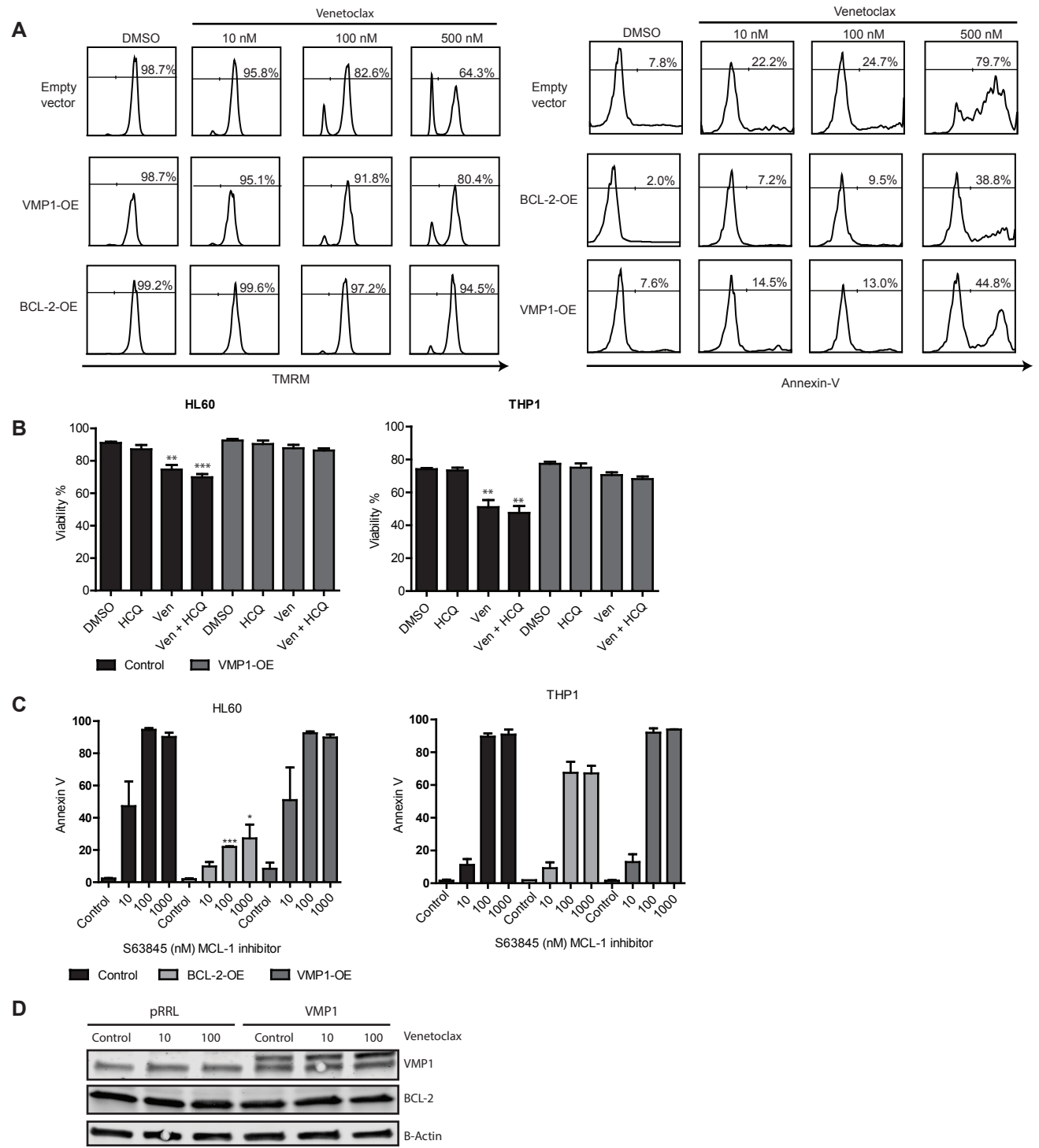

Supplement: Supplementary file 6 — Supplemental Figure S5 [file 41419_2019_1648_MOESM6_ESM.pdf]
